# Meningitis, and Its Treatment

**Published:** 1872-06

**Authors:** J. T. Jelks

**Affiliations:** Marietta, Georgia


### MENINGITIS, AND ITS TREATMENT.

BY J. T. JELKS, M.D., MARIETTA, GEORGIA.

In the discussions of the Atlanta Academy of Medicine, published in the ATLANTA MEDICAL AND SURGICAL JOURNAL for March, 1872, we find that Dr. John M. Boring treats meningitis with two and three grain doses of quinine, repeated every two hours. Dr. W. F. Westmoreland says he treats this disease with "quinine, opium, calomel, and a blister to the spine. He is dissatisfied with the treatment, as but a small per cent. of his cases recover."

Bearing in mind the pathological condition in this disease, let us analyze the above treatment.

First, let us take the quinine, and ascertain its physiological action on the system. Desiderio, in 1829, experimented with *small* doses, and concluded that quinine was a *stimulant*, and that this stimulation was "*hightened* by alcohol and *opium*, and lessened by cherry laurel-water, digitalis, and blood-letting." This conclusion has been confirmed by every physician who has used the drug in small doses, as evidenced by the brightened eye, animated face, buzzing ears, and vertigo. But Giacomini, experimenting with doses of forty and fifty grains, and noting only the sedative action on the heart, concluded it to be a sedative; and, in truth, it is a sedative, a very *powerful* one, but the sedation follows the *stimulant* action, just as depression follows the excitement produced by alcohol.

Magendie and Melier found that by giving to a dog one-half drachm of quinine, it produced poisonous effects, and was rejected by the stomach unless the œsophagus was tied; when this precaution was taken, death followed in twenty-four hours, with the occurrence of convulsions, dilated pupils, etc. On dissection, the lungs were found engorged with blood, and the vessels of the *brain* and stomach very considerably congested. (Stille Ther., vol. i., page 442.) Again (see same work, page 444), the salts of cinchona were administered to animals in large doses, producing death; and in *all* cases, "increased vascularity of the *brain* and *meninges*" was found on dissection.

We have seen that small doses of quinine are stimulating, and that large doses, after the stimulation has passed off, reduce the pulse in man; and that in animals, death follows with convulsions and dilated pupils; and that in all cases, the meninges and brain were very greatly congested. It is true, "a man is not a dog," yet the experiments upon the lower animals are very *pointed*, and especially so when they are sustained by the symptoms which manifest themselves in man while under the influence of this drug. Now, let the reader keep these facts in his mind, for we shall use them further on.

Take up opium, in the next place. The physiological effects of small doses—say one-fourth to one grain—are a brightened eye, enlivened eye, a reddened and suffused face. Let sleep come and pass, and tell me what means the *red*, congested eye, besotted face, and aching head? The effects of larger doses are but the hightening of the small ones. We find, by dissecting fatal cases, that the brain and meninges are greatly congested.

Bearing in mind the pathological conditions in this disease, and the physiological action of these two drugs, what good can *any* one *hope* to obtain from their exhibition? By the use of such a prescription, are you not adding *fuel* to the flame already kindled? Is it not *surprising* that even "a small per cent." of the Doctor's cases recover? Dr. Westmoreland would not dream of giving alcohol in the *active* stage of meningitis; then *why* employ quinine and opium? I can understand why it should be used in the *last stage*—when the inflammation has passed, and we must support the vital powers as much as we can—but I can see *no reason* in giving either *large* or small doses while the congestion is at its hight. In a case of pneumonia, where there is no *very* great desire or expectation of promoting the *exudation*, which acts as a local *depletant* to the distended capillaries, and whose natural tendency is to absorption, there can be no objection to the use of quinine and opium. But where we have the brain or its meninges inflamed, enclosed as they are by a firm and unyielding wall, we surely can not desire to *increase* the congestion, and thereby produce an effusion which, in this position, will kill our patient.

Having thus seen, from the physiological action of quinine and opium, that they should not be used in this disease, we come next to inquire, *What are the rational* indications of treatment? Evidently, our *first* endeavor should be to relieve the congestion, if possible. But if we are too late to check the inflammation and its results, then we must endeavor to sustain our patient, and, as far as we can, "*bridge* over" the stream that yawns to receive him.

To carry out the first indication, general blood-letting is very prompt, indeed, in a great many cases. If the pulse is hard and bounding, bleed your patient. Very frequently you do not find a hard, bounding pulse, but the very reverse—small, feeble, *oppressed;* and in these cases, blood-letting will often bring the *pulse up*, by reducing the *compression* of the brain produced by the great congestion. Standing high in the estimation of the profession, and *deservedly* so, we find veratrum viride and aconite. These, by their action on the heart, lower its force and frequency, thereby lessening the momentum and the quantity of blood sent to the brain.

Secondly, our object may be accomplished by those remedies which lessen the amount of blood in a given organ by stimulating *directly* the enlarged and paralyzed capillaries of said organ, thereby enabling them to return to their normal size—which done, the abnormal quantity of blood in said organ is removed.

In the valuable essay of Dr. Hammond, published in 1866, he remarks that bromide of potassium may "*almost always* be used with advantage to diminish the amount of blood in the brain." He further says: "I have administered it to dogs whose brains had been previously exposed to view by the trephine, and have *invariably* observed it to lessen the amount of blood circulating in the cranium, and produce *shrinking* of the brain as a consequence. Moreover, we have only to observe its effects in the human subject, to be convinced that this is one of the most important results of its employment. The flushed face, throbbing carotids and temporals, the suffusion of the eyes and fulness of the head, disappear as if by magic under its use." Dr. Alfred Stille, after enumerating the physiological effects of the bromide of potassium, as observed by Voisin, says: "All of these symptoms, *without exception*, appear to be attributable to the power possessed by the medicine of *restraining* the capillary circulation of the nervous centers *chiefly*." If these things be true, and no one can disprove them, then where will you find so valuable a remedy for the congested state of meningitis as the bromide of potassium. True, it may sometimes disappoint us, but we do not claim for it the virtues of a *specific* for this disease, or indeed any other.

The congestion of the brain may be, in a measure, relieved by those remedies which act as derivatives. For this purpose, we may use powerful cathartics, as croton oil, podophyllin, etc. Sinapisms and blisters are *very* useful; but to be beneficial, they must be *extensive*, thereby setting up a controlling center of irritation away from the brain, carrying out the old maxim, "*Ubi irritatio ibi fluxus*." Should these means fail to arrest the disease in its first stage, and collapse supervene, *then* are we justified in using quinine or alcohol.

I do not deem it necessary to go into the minute details of cases, and will merely state, that in the spring and summer of 1869 I saw two cases, in the practice of Dr. J. E. Cook, Culloden, Monroe county, Georgia, treated with large doses of bromide of potassium, an extensive blister to the neck and spine, giving at the same time a free mercurial purge, in the first stage; in the second, iodide potassium, quinine, and nutritious food—the quinine not being open, in this stage of the disease, to the objection of increasing the congestion, *that* having passed away. I have treated three cases, these three and those of Dr. Cook all recovering.

P. S.—Since writing the above article, I have attended the medical convention of this State, and these views were fully confirmed by the analysis of eighty-eight cases reported by Dr. Griggs, of West-Point—his first nine cases all dying; the remaining seventy-nine recovering, if I remember correctly, reated by the lancet, bromide of potassium, and the blister, and *when the patients required it*, food and tonics. These cases are in very great contrast with the small per centum of recoveries in the practice of Dr. Westmoreland.

